# Statistics and geometry of orientation selectivity in primary visual cortex

**DOI:** 10.1007/s00422-013-0576-0

**Published:** 2013-11-19

**Authors:** Sadra Sadeh, Stefan Rotter

**Affiliations:** Bernstein Center Freiburg, Faculty of Biology, University of Freiburg, Hansastr. 9a, 79104 Freiburg, Germany

**Keywords:** Orientation selectivity, Orientation map, Columnar structure, Random connectivity, Primary visual cortex

## Abstract

Orientation maps are a prominent feature of the primary visual cortex of higher mammals. In macaques and cats, for example, preferred orientations of neurons are organized in a specific pattern, where cells with similar selectivity are clustered in iso-orientation domains. However, the map is not always continuous, and there are pinwheel-like singularities around which all orientations are arranged in an orderly fashion. Although subject of intense investigation for half a century now, it is still not entirely clear how these maps emerge and what function they might serve. Here, we suggest a new model of orientation selectivity that combines the geometry and statistics of clustered thalamocortical afferents to explain the emergence of orientation maps. We show that the model can generate spatial patterns of orientation selectivity closely resembling the maps found in cats or monkeys. Without any additional assumptions, we further show that the pattern of ocular dominance columns is inherently connected to the spatial pattern of orientation.

## Introduction

### Orientation selectivity

The function of cortex can be studied by looking at its functional properties that first emerge at this level and that are absent in more upstream brain structures. Orientation selectivity (OS) is paradigmatic in this respect: Many neurons in the primary visual cortex (V1) of mammals respond selectively to oriented stimuli (Hubel and Wiesel 1962, 1968; Niell and Stryker 2008) while they are receiving thalamic input from neurons in the lateral geniculate nucleus (LGN), with almost no selectivity for orientation. Being simple and tractable, this sensory feature has provided a framework for studying structure and function of sensory areas in the mammalian brain for many decades now (Ferster and Miller 2000; Sompolinsky and Shapley 1997).

**Fig. 1:**
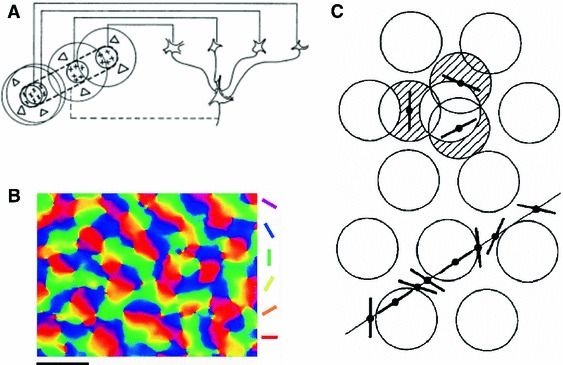
Orientation selectivity and orientation map. **a** Feedforward alignment of receptive fields underlying the elongation of cortical receptive fields in simple cells as suggested by Hubel and Wiesel (1962). Circular receptive fields of LGN neurons connected to a simple cell in V1 are aligned with a specific orientation, which makes the cortical receptive field elongated. **b** Orientation map in monkey striate cortex revealed by voltage-sensitive dye imaging. *Scale bar* has length $1\,{\mathrm {mm}}$. Modified from Blasdel and Salama (1986) (Swindale 2008). **c** Hexagonal grid of columns in a model by Braitenberg (1985). At the center of each white circle, an inhibitory population was conjectured, which would inhibit the response of neurons at the orthogonal orientation. Both orientation selectivity of neurons as a result of their position with respect to columns (*upper*, indicated by *bars*) and their succession as one moves horizontally on the surface of the cortex (*lower*), are implied by the model (all figures reproduced with permission)

Hubel and Wiesel (1962) themselves provided the first structural explanation for the emergence of OS in simple cells. Their argument was based on a feedforward alignment of receptive fields, where ON and OFF center LGN cells were connected to the ON and OFF subregions of a simple cell in V1, respectively, leading to an elongation of cortical receptive fields (Fig. 1a). Later experimental studies, which mapped the receptive field of neurons using the method of reverse correlation, indeed confirmed that there actually is such a feedforward match (Tanaka 1983; Reid and Alonso 1995).

However, the following general question still needs to be answered: Which basic mechanism underlies the formation of this structure? Is it an inborn property of the visual system (“nature”), or is it acquired by experience (“nurture”)? At one extreme, orientation selectivity might be genetically encoded: Genetically encoded markers would guide thalamocortical axons to wire up such that the appropriate receptive fields emerge. This would then provide the animal with optimized feature detectors to survive in its natural environment. At another extreme, orientation selectivity might be learned through visual experience: Orientation selectivity would not be hardwired, but a result of adaptation during brain development, driven by the statistics of the natural sensory inputs.

Conceptually, a model of the latter sort would provide an attractive scientific explanation: It suggests a general mechanism, which could readily be extended to other modalities; the former scenario, in contrast, renders OS a unique feature of the visual system. Activity-dependent models that exploit correlations are one specific example of such models. These models are also more amenable to experimental tests, as they demand a specific pattern of activity and correlations for the establishment of selectivity during development. They rely on patterned visual input and may therefore predict that OS would not develop in the absence of visual stimulation. Developmental studies, however, have reported OS in young animals that lack any visual experience (Hubel and Wiesel 1963). Although the maturation of OS depends on visual experience, its initial establishment appears not to depend on the pattern of visual stimulation (Albus and Wolf 1984; Chapman and Stryker 1993; Löwel et al. 1998).

Theoretically, it is still possible, however, that the pattern of correlations in spontaneous activity instructs the formation of oriented receptive fields (Miller et al. 1999). A model based on this would, therefore, need a specific pattern of correlations in the spontaneous activity during development. In the model by Miller (1994), this is a Mexican hat-like pattern of correlations, where nearby inputs of the same sign (ON or OFF) are best correlated, while ON center inputs are best correlated with OFF center inputs at larger distances. This pattern, however, is not supported by experiments (Ohshiro and Weliky 2006; Weliky and Katz 1999).

### Statistical connectivity

Is there an alternative explanation? There is indeed a family of models based on “statistical connectivity,” which resides in the middle between the two extremes of “nature” and “nurture.” These models take the statistical pattern of cortical wiring as their premise and try to come up with an interpretation of the function in terms of connectivity. Valentino Braitenberg was indeed pioneering an approach where statistics and geometry of neuronal connectivity is the only constraint of a model (Braitenberg and Schüz 1998). The idea is that a generic pattern (like, for instance, random connectivity) would enable the system to serve its function.

The relevant connectivity in the case of OS is the pattern of divergence and convergence between LGN and cortex (V1). Here, divergence means that each individual LGN neuron projects to many neurons in V1. This, in turn, implies a high convergence of inputs from LGN to a V1 neuron, that is, each individual V1 neuron receives input from many LGN neurons (Peters and Payne 1993). In view of the lateral extent of this divergence, a huge overlap of axonal arborizations is also implied for LGN afferents (Peters and Payne 1993). This type of connectivity has been considered in a more recent model based on “haphazard wiring” (Ringach 2004, 2007). It was shown that such a random pattern of convergence from thalamus to cortex, in addition to the mosaic of ON and OFF center retinal ganglion cells on the retina, could explain the emergence of simple cell-like receptive fields.

There are, however, problems with this model. One problem is that the number of LGN neurons converging to a V1 cell in the model is smaller than the real values that were estimated to be in the range 30–100 (Alonso et al. 2001; Peters and Payne 1993). Also, the degree of elongation of receptive fields in the model does not match that of V1 simple cells (Ringach 2004). Moreover, it is not clear how the two patterns of OS established by the two eyes can be brought to match on the cortical surface, as each one is established by a different retinal mosaic. It is possible to invoke some sort of activity-dependent plasticity, which aligns the topography of OS induced by the ipsilateral eye with the already established contralateral map (Ringach 2004). But this raises again the question to which extent a developmental mechanism is involved in the establishment of OS, and why almost the same structure is established when the animal is stimulated by either eye (Gödecke and Bonhoeffer 1996).

All these problems seem to be related to the fundamental assumption made in the model that OS is essentially a retinal property. Indeed, simple cells in this model are generated by sampling from a handful of ON and OFF center retinal ganglion cells, reflecting the features relayed to them from the periphery. As a result, the degree of convergence must be limited in order not to distort the retinal seed of selectivity, the elongation is limited to the aspect ratio of ON–OFF dipoles on the retina (Paik and Ringach 2011), and the structure of OS inherited from each eye can be totally different. All together, the model deviates from the concept of OS as an emergent property of the cortex. In fact, two radically different schemes might emerge from here: The first one argues for OS as a feature that is already determined at the level of retina, but manifests itself only in the cortex, where ON and OFF center channels meet for the first time; the second one argues that OS is due to an elongation of receptive fields, which is a result of the convergence of many inputs with non-elongated receptive fields, and which makes the receptive fields of cortical neurons larger than those of their retinal or thalamic afferents.

### Orientation map

Hubel and Wiesel (1968) also described the topographic organization of OS in the cortex. Penetrations perpendicular to the cortical surface revealed a “columnar” organization, where all neurons encountered showed a similar preferred orientation. The same was reported in the somatosensory cortex (Mountcastle 1957), where all neurons in a column shared similar features. The map of selectivity on the surface of cortex was, however, different: Unlike the somatosensory cortex, no discrete columns were found. Tangential penetrations encountered a smooth and continuous progression of preferred orientations, although some sudden transitions were also observed (Hubel and Wiesel 1974). Later, optical imaging studies revealed a large-scale organization of these maps (Blasdel and Salama 1986; Ts’o et al. 1990; Bonhoeffer and Grinvald 1991): They exhibit an orderly arrangement of OS, where neurons with similar selectivity tend to cluster in iso-orientation domains (Fig. 1b). The transition between different domains can be smooth in linear zones, but there are also discontinuities where different selectivities occur next to each other (Obermayer and Blasdel 1993). One specific form of discontinuities is the “pinwheel” centers, singularities around which all orientations are represented once, either in clockwise or in counterclockwise fashion.

Explaining origin and function of this particular organization has indeed attracted much attention from both experimenters and theoreticians (Swindale 1996). As in the case of OS, there is evidence in favor of “nature” models: Experimental findings suggest that the overall layout and geometry of the orientation map is established very early during development and remains unchanged during the rest of the developmental period (Chapman et al. 1996; Gödecke et al. 1997; Löwel et al. 1998; Sengpiel et al. 1998). The orientation map is indeed present even in animals lacking any visual experience (Wiesel and Hubel 1974).

The correlation-based model of OS mentioned above is one of the models which has been suggested by theoreticians (Miller 1994). As the model relies on the structure of correlation among neighboring neurons, it cannot explain the reported heterogeneity of nearby neurons in their spatial phase (DeAngelis et al. 1999). An alternative explanation is provided by a model based on statistical connectivity (Ringach 2004, 2007; Paik and Ringach 2011). As in the case of OS, the model suggests the retina as the origin of the orientation map: The continuity of the map comes from the fact that neighboring cortical neurons share input from the same retinal cells (Ringach 2004), and the periodicity arises from a Moiré interference pattern of the hexagonal grid of ON and OFF retinal ganglion cells (Paik and Ringach 2011). Recent studies, however, have demonstrated that it is unlikely that such retinal mosaics drive the formation of cortical orientation maps (Hore et al. 2012; Schottdorf et al. 2013). Also, as discussed above, the question of how two independent monocular maps are eventually brought to match on the cortical surface is not answered by the model (Ringach 2004), unless it is complemented by a developmental mechanism. Moreover, it would be difficult for the model to explain how identical orientation maps would develop for both eyes without common visual experience (Gödecke and Bonhoeffer 1996), since the mosaic of retinal ganglion cells for each eye and the interference pattern between the ON and OFF grids would be different. Again the model cannot account for these observations, since it is based on the geometry of retina. The constancy of maps, however, seems to favor an explanation based on the geometry of cortex.

### Geometry of cortex and organization of orientation

There is a theory of OS and its organization based on the geometry of cortex (Braitenberg 1985, 1992; Braitenberg and Braitenberg 1979; Braitenberg and Schüz 1998). In this model, both the orientation specificity and the organization of receptive fields are determined by the geometry of cortical columns. The model is based on the experimental results by Hubel and Wiesel (1974) and postulates that orientations are organized in “hypercolumns,” each hypercolumn containing a full set of all orientations, arranged around a center. It is assumed that at the center of each hypercolumn exists a population of inhibitory neurons, which suppresses the activity of cortical neurons at orthogonal orientations. Therefore, the response would be the strongest, if an elongated visual stimulus was properly located at an orientation such that the neuron is optimally activated by the bar, but not inhibited by the central inhibitory population. This geometric arrangement also determines the OS maps on the cortical surface (Fig. 1c).

Braitenberg’s model is simple and appealing, and it can potentially explain many properties of the spatial organization of OS. However, there are several issues with the model (Braitenberg and Schüz 1998). The first issue is concerned with the size of receptive fields: Real receptive field sizes are larger than what the model suggests, about two or three times the size of a hypercolumn. To resolve this discrepancy, the authors of the study invoke “composite receptive fields” of cell assemblies. They argue that the receptive fields experimentalists measure in real cortex are not those of a single neuron; rather they belong to a cluster of pyramidal cells with similar response properties. These clusters are responding as a whole as if they were wired together by a Hebbian connectivity rule; this, in turn, increases the size of their composite receptive field. The problem with this suggestion is that it would again raise the question if, and to which extent, developmental mechanisms contribute to the process of receptive field formation and maturation. A more parsimonious model would account for this fact without appealing to learning. From an experimental point of view, however, the issue needs further experimental investigation, particularly by reporting the size of receptive fields during development.

Another issue with the model is the structure of pinwheels it predicts. If pinwheels are located at the center of hypercolumns, the model predicts that each orientation is represented twice when circling around each of these singularities. This feature is not consistent with experimental pinwheels, around which each orientation is only visited once. Braitenberg (1992) has made the point, however, that the actual pinwheels are not located at the center of hypercolumns but, instead, appear between hypercolumns (Valverde and Braitenberg 2007a, b).

There is, however, still an issue in interpreting hypercolumns with respect to ocular dominance columns (ODCs). If hypercolumns can be identified with ODCs (as we assume later in our model, see Sect. 2), the appearance of pinwheel centers between hypercolumns is incompatible with experimental findings: In cats, for example, a strong correlation between pinwheel centers and ODC centers has been reported (Crair et al. 1997a, b). This problem seems even more serious in monkeys, where the relationship between pinwheels and ODCs is more pronounced: Orientation pinwheels appear to be strictly avoiding the borders of ODCs and, in fact, tend to lie on ODC midlines (Obermayer and Blasdel 1993). As in Braitenberg’s model no relationship between hypercolumns and ODCs is necessary, it is conceivable that some specific configuration of hypercolumns with respect to the ODC pattern beyond the model can explain it. It might, however, be difficult for the model to account for some further constraints: In Braitenberg’s model, it is assumed that the inhibitory populations are localized within cytochrome oxidase-rich regions (CO blobs; see also below), and CO blobs indeed appear at the center of ODCs (Horton and Hubel 1981).^1^

Last but not least, the existence of the presumed “lumped inhibitory neurons” at the center of columns has not been corroborated in experiments. It has been suggested that, out of several kinds of GABAergic neurons that have been reported to exist in cortex, one group might be found to have a higher concentration in ODC centers and a group has indeed been identified (Braitenberg and Schüz 1998; Jones et al. 1994), which is preferentially localized within the CO blobs. Given the role of inhibitory neurons in the model, a specific arrangement of them is also needed, with their dendrites in the hypercolumn centers, and their axons emanating radially to reach the surrounding pyramidal cells (Braitenberg and Schüz 1998). Whether the specific presence and arrangement of clustered inhibition is supported by experiments awaits further research.

In the following, we propose an alternative model that we think is more consistent with biological data, while at the same time preserves the essential features of the geometric model. We complement the geometric model by taking the statistical connectivity of thalamocortical afferents from LGN to V1 into consideration and propose that this approach provides a better explanation for the emergence of feature maps.

## Results

### Columnar receptive field

In layer IV cat primary visual cortex, afferent connections from a single LGN neuron terminate and ramify in a region of approximately $1\,{\mathrm {mm}}^2$ (Peters and Payne 1993). In our simplified model, we assume that the arborization has the same size for all LGN cells, neglecting the fact, among other things, that y-cells have larger termination areas than x-cells. Also, we do not include different center-surround types of LGN neurons in our model; we take all LGN cells to be of the ON center type.

A “column” in our model is the aggregate arborization of many LGN afferents with similar receptive fields. Thus, we do not explicitly consider here the differences in size and shape of LGN receptive fields (Chapman et al. 1991; Jin et al. 2011). This columnar organization reflects the ocular clustering of afferents very early in development, which already looks like the organization of mature ocular dominance columns (Crowley and Katz 2000). As Katz and Crowley (2002) put it: “Axons initially grow to their correct locations and generate increasingly dense arborizations, with little evidence of overlap between adjacent columns.”^2^ Note that this columnar structure is purely a property of thalamocortical projections and does not imply (nor does it contradict) any columnar structure in the recurrent wiring of cortex. Indeed, in our model, we assume no clustering of cortical neurons and model them as a homogeneous network in a two-dimensional plane corresponding to the cortical surface (see below).

**Fig. 2:**
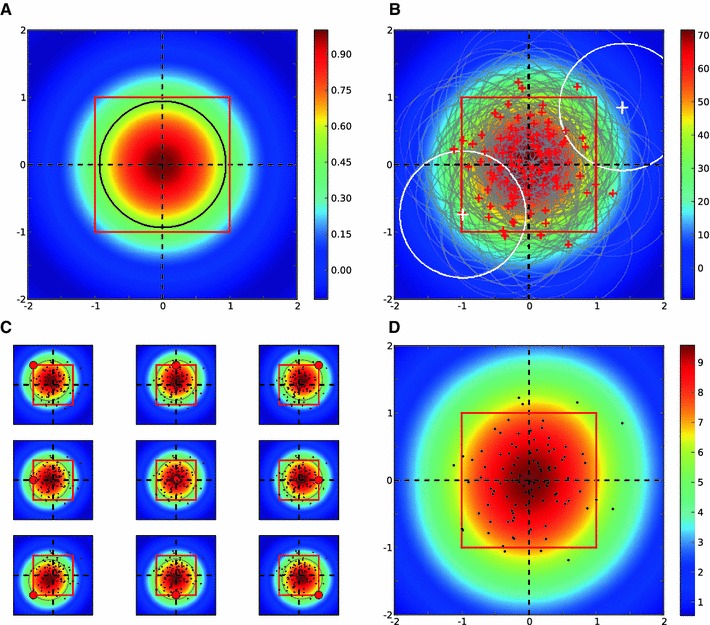
Columnar receptive field. **a** Receptive field of an LGN afferent, modeled as a normalized difference of Gaussian with $\sigma _\mathrm{on} = 1$ and $\sigma _\mathrm{off}=1.5$. *Black line* indicates the contour of half-maximum height (0.5). **b** Receptive fields of all $N = 100$ LGN afferents projecting in one column, as assumed in the model. *Red crosses mark* the LGN centers (the standard deviation of distribution is $\sigma _c = 0.5$; see Sect. 4), and the *gray lines* are the half-height contours in each case. The *colors* code the sum of all individual receptive fields. Two *white crosses* and *white circles* show, respectively, the centers and half-height contours of two LGN receptive fields with the maximum distance. This distance is 2.85. **c** Receptive fields in our model, for cortical neurons at different positions on the column (denoted by the *small circle* in *red*). *Dots* show the centers of afferents. $\sigma _w = 1$. **d** The peak value of the cortical receptive field at each position on the column. *Dots* shows the centers of afferents

We model the receptive field of LGN neurons as a difference in Gaussians (Fig. 2a). These receptive fields are highly overlapping for all LGN neurons within a column, as they cover a similar area in the visual field (Fig. 2b). The centers of these receptive fields have a Gaussian distribution approximately aligned with the center of the column. The density of axonal arborizations of each LGN afferent is also assumed to have a Gaussian distribution.

We combine all these Gaussians to obtain an aggregate receptive field that describes the input that each cortical neuron receives from the column. This is a weighted sum of all LGN receptive fields; the weights are given by a Gaussian function of the distance to each position on the cortical surface. This is a consequence of the Gaussian distribution of axonal termination points, which implies a higher connection probability for close-by cortical neurons. Simulated samples of this aggregate receptive field for different cortical positions are shown in Fig. 2c. Since this is a reduced receptive field, which summarizes the total effect of all afferents within a column, we call it the “columnar receptive field.”

Columnar receptive fields are larger than the receptive fields of individual LGN neurons. This is a result of many partially overlapping receptive fields. Moreover, as a result of the massive overlap, receptive fields are very similar for different cortical positions with respect to the column, as shown in Fig. 2c. This fact justifies that we reduce the complexity of a column and describe it by an aggregate receptive field, as introduced here. This means, for all neighboring neurons, the effect of each column is now reduced to the columnar receptive field inherited from it. The weight of this columnar receptive field for each cortical neuron depends on its distance to the center of the column and can be approximated by an effective Gaussian (Fig. 2d).

**Fig. 3:**
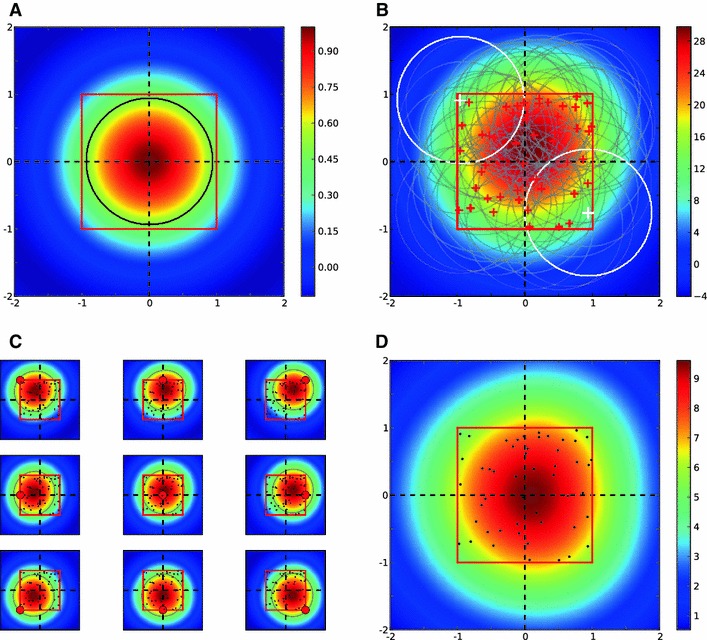
Columnar receptive field for a uniform distribution of LGN terminals. **a**–**d** Same as in Fig. 2, but for a uniform distribution of LGN centers. $x$ and $y$ positions of LGN centers (*red crosses*) are taken randomly from a uniform distribution between $[-1, 1], N=50$. Other parameters and conventions are the same as in Fig. 2

**Fig. 4:**
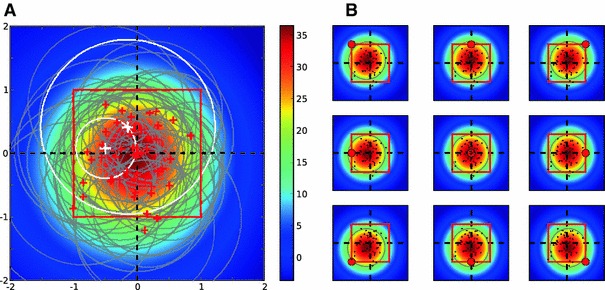
Columnar receptive field from heterogeneous LGN receptive fields. **a** $N = 50$ receptive fields of LGN afferents projecting to one cortical column, as in Fig. 2b. Unlike Fig. 2b, the sizes of receptive fields are now not the same: $\sigma _\mathrm{on}$ has a uniform distribution between $[0.5, 1.5]$, and $\sigma _\mathrm{off}=1.5 \sigma _\mathrm{on}$ in each case. The half-height contours for the smallest and the largest receptive field are shown in *white*. The *colors* code the sum of all individual receptive fields. **b** Cortical receptive fields, for neurons at different positions in the column (denoted by the *small circle* in *red*). *Dots* show the centers of afferents. $\sigma _w = 1$

We use this simplification in the following to highlight the role of columnar interaction for orientation selectivity. Note, however, that some of the assumptions can be further relaxed. First, it is not necessary to assume a precise Gaussian distribution of inputs from LGN around the center of columns. In fact, allowing for a uniform distribution of LGN receptive fields in the column results in similar columnar receptive fields, only slightly shifted from the center (Fig. 3). Second, sampling from heterogeneous LGN receptive fields with different sizes does not change the result qualitatively (Fig. 4). Finally, the concept of a columnar receptive field itself is not a strictly necessary assumption of the model; we also consider a more general scenario without this assumption later.

### Size of receptive fields

**Fig. 5:**
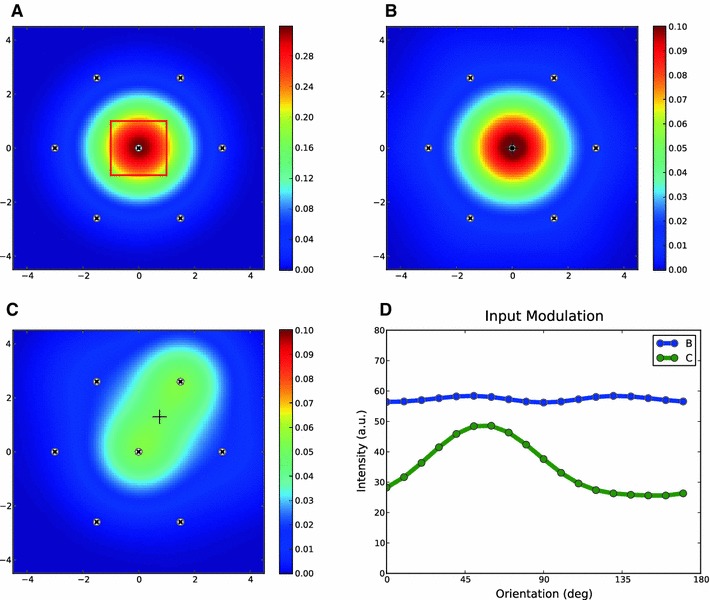
Multi-columnar receptive fields. **a** A hexagonal grid of columns described in Fig. 2. Shown is the aggregate receptive field of the center column. Other columns have the same columnar receptive fields, centered at the center of columns (*small white circles*). **b** Receptive field of a neuron located at the center of a central column. **c** Receptive field of a neuron located between the central column and one of the neighboring columns. **d** Tuning curve of neuronal input. The receptive field of the neuron is stimulated with drifting gratings of 18 different orientations (shown on the *x*-*axis*). The temporal modulation of the response for each orientation is shown on the *y*-*axis*

The simplification introduced in the previous section allows us to go beyond one column in our model and investigate interactions between columns. Let us first consider a hexagonal grid of such columns (Braitenberg 1985), as shown in Fig. 5a. The receptive field of each cortical neuron in this columnar structure is given by a weighted sum of all the columnar receptive fields. The corresponding weights come from a Gaussian function of the distance to the center of each column.

The receptive fields obtained in this manner are larger than each individual columnar receptive field, since a cortical neuron sees several columns, depending on its position in the columnar grid. If a neuron is located close to the center of a column, the within-column density of branches is so dominant that the receptive field is mostly determined by this particular column. Shown is the case of a neuron exactly at the center in Fig. 5b. In contrast, a neuron which is located exactly in the middle between two columns would see both columnar receptive fields with the same weight and, as a result, have itself a larger receptive field (Fig. 5c). An increase in size of the receptive field is not the only consequence, however. As a result of the elongation in its receptive field, the neuron also attains orientation selectivity.

### Orientation selectivity

Depending on the position of a cortical neuron relative to the grid of cortical columns, it would integrate the columnar receptive fields differently. As a result, the exact shape of receptive field elongation, and hence the orientation selectivity that follows from it, varies across neurons. If a neuron is located at the center of a column (as in Fig. 5b), no elongation of its receptive field results, as a single, isotropic columnar receptive field is dominating it. In contrast, if a neuron is located between two columns, it sees effectively two neighboring columnar receptive fields, which leads to an elongation of its combined receptive field. This neuron now becomes orientation selective, responding best to a stimulus orientation of the line connecting the two columns (Fig. 5c). Note that, although there are no clusters of inhibitory neurons in the column centers, the perfect symmetry of the columnar structure in combination with the columnar receptive fields effectively “inhibit” orientation selectivity in the center of columns. The reason is that the highest density of afferent arborization in the center makes the isotropic, non-oriented receptive field of the column dominant, preventing any receptive field elongation.

To quantify orientation selectivity at each position, we stimulate the system with drifting gratings of different orientations. In the absence of nonlinearities, the scalar product of the grating with the receptive field of any neuron predicts the net feedforward input to this neuron. Neglecting recurrent interactions, this also determines the membrane potential response, temporally modulated at the frequency of the stimulus. We take the temporal F1 component as a measure of anisotropy of response, similar to what experimentalists do in intracellular recordings, when they extract orientation selectivity from the modulation component of the responses (Ferster et al. 1996; Carandini and Ferster 2000).

We compute the F1 component at different orientations and this way obtain a tuning curve of the neuron. This is shown in Fig. 5d for the two receptive fields discussed before (Fig. 5b, C). The elongated receptive field implies a clear modulation across different orientations, while the tuning curve for an isotropic receptive field is flat. We take the orientation of the strongest response as the preferred orientation (PO) of the neuron. To quantify the degree of selectivity, we employ an orientation selectivity index (OSI), which amounts to $1 - {\mathrm {circular~variance}}$ of the tuning curve (see Sect. 4).

**Fig. 6:**
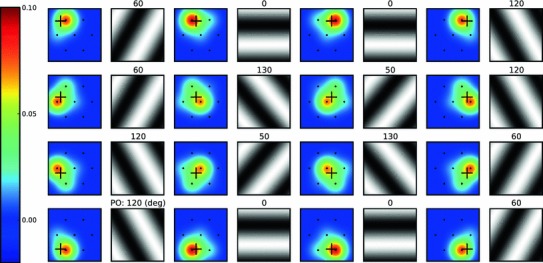
Sample receptive fields from the columnar grid. For 16 neurons at different positions on the hexagonal grid (Fig. 5), the resulting receptive field is plotted on the *left*. The evenly sampled positions are shown by *crosses*. For each receptive field, the best-matching grating (i.e., the grating at PO) is shown on the *right*

Figure 6 shows the result of this procedure for some sample receptive fields. Receptive fields obtained at different cortical positions are shown along with the best-matching stimulus, respectively. There are different degrees of selectivity, ranging from almost isotropic to strongly elongated, and for all examples shown the PO is matching the one inferred by visual inspection very well.

### Pinwheels and spatial organization of orientation selectivity

**Fig. 7:**
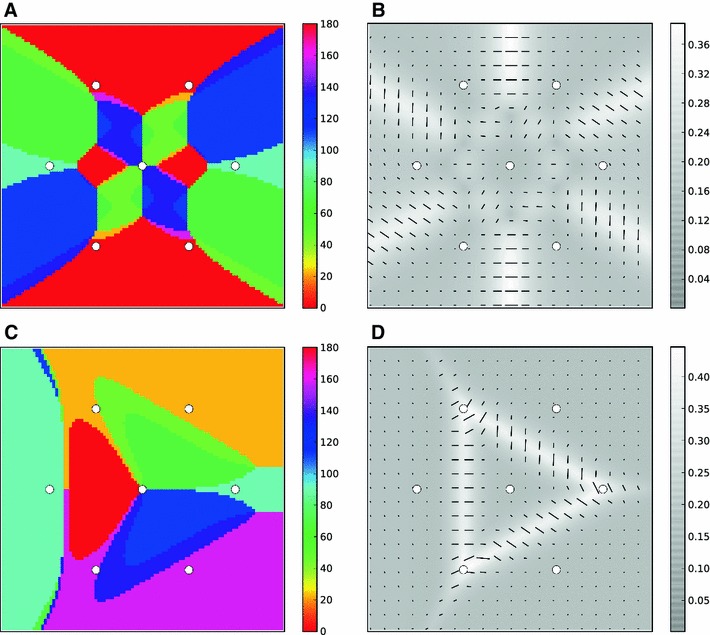
Map of orientation selectivity for the columnar grid. **a** A pseudocolor map of orientation selectivity, which shows the PO of neurons at different positions on the columnar grid. *White circles* depict the center of columns. **b** A map of orientation selectivity, where the PO at each point is represented by the orientation of the marker line. Its length is proportional to the degree of selectivity (OSI). A map of selectivity (brighter regions denoting higher OSI) is shown in *the background*. **c**, **d**) Same as (**a**, **b**) for the monocular grid. Half of the surrounding columns are deleted

Once we have obtained PO and OSI of cortical neurons, we can also study the properties of the map of orientation selectivity that emerges on the cortical surface. Figure 7a depicts this map for the hexagonal grid of columns discussed in the previous section, where the PO of each neuron is indicated by a hue value. The maps obtained in this way indeed have continuous regions of selectivity (iso-orientation domains) as well as pinwheel-like singularities. Singularities coincide with the centers of columns, where the OSI is in fact zero (Fig. 7b), and where all orientations are represented in a small neighborhood around the center. However, not unexpectedly, each orientation is represented twice in each pinwheel, implied by the geometry of the hexagonal grid.

The same problem arises in Braitenberg’s model, if pinwheels are localized at the centers. He argues, however, that the visible pinwheels are not localized at the hypercolumn centers. Rather, they emerge between hypercolumns (Braitenberg 1992). The region in the hypercolumn center (as opposed to the pinwheel center), he argues, is not orientation selective, and this lack of selectivity is masked by the way data obtained by optical imaging are processed for display. Also, since the between-hypercolumn pinwheels are more numerous in a hexagonal structure, they are more visible in an orientation map. Hypercolumn centers are also present in these maps, however, but their identification needs a closer look.

As discussed above, the apparent coincidence of pinwheel centers with the centers of ocular dominance columns may argue against this interpretation. Although pinwheel centers do also appear between hypercolumns, they could as well be present close to the column centers. We explain here how this happens in our model, i.e., how pinwheel centers can be localized at the center of columns without seeing each orientation twice.

In binocular animals, the cortex needs to accommodate columns of LGN afferents from both eyes. Assuming that each column is essentially dominated by one eye (ocular dominance columns), monocular maps would deviate from binocular maps. In our simple model based on a hexagonal grid, one way to account for this is to assume alternating ocularities. In monocular maps, the binocular hexagonal grid would then be reduced to a triangular neighborhood, with three columns of the same ocularity around the central column (Fig. 7c). If we now compute the orientation map for this reduced structure, there would still be a pinwheel in the center, but each orientation is only represented once around each singularity (Fig. 7c, d). Note that the remaining columns belonging to the opposite ocularity would have their own pinwheel center at a different position. This position is determined by the relative geometry of monocular columnar projections. As a result, two $180^\circ$-pinwheels (pinwheels with each orientation appearing once around them) are now born out of one $360^\circ$-pinwheel (pinwheel with each orientation appearing twice around it).

### Ocular dominance columns and orientation maps

For a more realistic version of the model, we will now consider more realistic patterns of ODC. In fact, given any ODC pattern, our model would eventually predict the corresponding orientation map. The only parameters then are the extent of columnar receptive fields and the connection weights.

There are, in fact, different patterns of ODC observed for different species, ranging from a patchy structure as incats (Löwel and Singer 1987) to stripes in monkeys (LeVay et al. 1975). From a theoretical point of view, different patterns of ODC might simply reflect different strategies to solve one problem: To maintain retinotopy simultaneously for two eyes. The problem is to tile a two-dimensional sheet (cortical surface) with ocular dominance columns, while keeping the proximity of their receptive fields on the retinal grid. This is not a trivial problem, since each column on the cortical grid must now maintain the neighborhood of input from both eyes. As a result, distortions of retinal coordinates are inevitable.

We now include these aspects into our model by considering two different coordinate systems, one for the visual field and one for the cortical surface. From the centers of receptive fields on the 2D “retinal grid,” afferents from each eye are projected (via the LGN) to cortex in a columnar fashion; this in turn forms the “columnar grid,” as discussed previously.

If columns were completely binocular, the same retinotopic mapping from the two eyes to the columns could be assumed. This is obtained by sampling from the same position in the visual field for both eyes and directing afferents of both ocularities to each (binocular) column. If columns were monocular, in contrast, the same retinal grid would be mapped to two separate columnar grids, one for each eye, respectively.

Whatever the precise ODC structure is, we assume that the centers of columns (for both ocularities) are maintaining a good match with the centers of the retinal grid, in order to preserve the topography of the retinal projection as much as possible. The competition between left and right eye for their grid positions on the same cortical surface can only result in a displacement from the best-matching pattern of monocular projections.

**Fig. 8:**
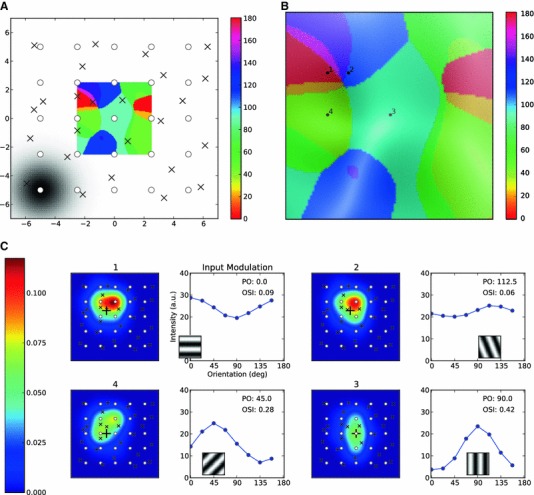
Orientation map of a displaced grid. **a** The retinal grid (*white circles*) and the columnar grid (*black crosses*) are shown together. The retinal grid is a regular Cartesian grid. The columnar grid is obtained by applying a random displacement (uniformly sampled from $[-1.5,1.5]$) to each coordinate of all grid positions. A sample columnar receptive field is shown on the *lower left*. Other columns have the same extent of receptive fields. Note that the receptive field is given in retinal coordinates. The resulting map of PO for the columnar grid is shown for the central region (to avoid boundary effects). Note that this map is displayed in columnar coordinates. **b** The same orientation map as in **a**. The OSI for each position is shown as the *brightness of the color*, with *brighter colors* corresponding to higher selectivity. **c** Four sample receptive fields from different locations on the map. The crosses show the position of sampling, corresponding to the numbers denoted in **b**. The tuning curves of input modulation, along with the best-matching gratings and the values of PO and OSI, are shown on the *right* in each case

In theory, one way to implement this scheme is to first make the columnar grid an identical copy of the retinal grid, assuming perfect retinotopy. For simplicity, we are neglecting here different cortical magnification factors for the center and the periphery. Then, we randomly displace the positions of cortical columns to mimic the displacement due to the competition of two eyes. The randomly displaced copy of the retinal grid now represents a monocular columnar grid. The result of such a procedure for the projections from one eye is shown in Fig. 8a. Note that we have now relaxed the assumption of hexagonal structure of columns and, instead, placed the column centers on a square lattice for the retinal grid. The model, therefore, does not depend on the specific arrangement of the columns.

We then assign a columnar receptive field to each column, as explained before. The receptive field of a neuron at a given position on the cortical surface is, as before, obtained as a weighted sum of columnar receptive fields, with weights being a function of the distance to each column center. For each receptive field, we then compute the PO and in this way construct the full orientation map. A monocular map of orientation (obtained from the central region to minimize boundary effects) is also shown in Fig. 8a.

Zooming into the map (Fig. 8b) reveals iso-orientation domains as well as characteristic singularities (pinwheels). Note that, although the displacement between retinal and cortical grids is small, the resulting map is quite different from a regular map that would be expected from a regular lattice. Moreover, pinwheels are now structured like the pinwheels in Fig. 7c, i.e., each orientation is represented only once around its center. Near these singularities, the selectivity is low. This is indicated by a saturation map superimposed on the image, with brighter colors denoting higher selectivity. The most selective regions are the middle parts of the iso-orientation domains. The four sample receptive fields and their tuning curves shown in Fig. 8c indicate all the same trend: Neurons closer to pinwheel centers are less selective, as their tuning curves are less strongly modulated. Neurons in the center of iso-orientation domains, in contrast, exhibit stronger tuning, since the elongation of their receptive fields is more pronounced.^3^

### Relaxing columnar receptive fields

We used the simplified model in the previous sections to illustrate how orientation selectivity and realistic orientation maps could be obtained from the columnar pattern and the statistics of thalamocortical connectivity. The reduced columnar receptive field that we introduced served this illustrative purpose and made the model conceptually and computationally simpler. In this section, we now introduce a similar columnar model without resorting to an aggregate receptive field for each column. The model is more efficient for numerical simulations, which allows us to more conveniently explore the properties of larger orientation maps.

**Fig. 9:**
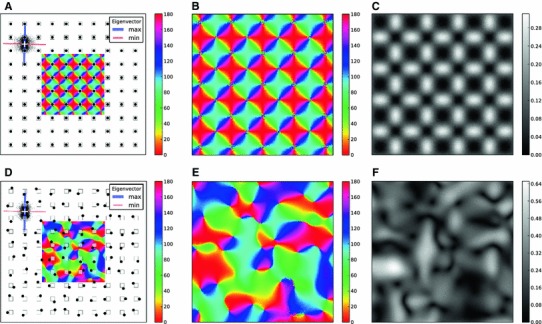
Orientation map of a larger grid. **a** The retinal and columnar grid points are marked by *white squares* and *black circles*, respectively. For a given position on the grid, an ensemble of $n = 10,000$ positions are randomly drawn, depending on the position of the neighboring columns. The orientation of the receptive field is given by the axis of elongation of the ensemble (see Sect. 4 for details). The eigenvectors corresponding to the maximum and minimum eigenvalue are shown by the *blue* and the *red bars*, respectively. The width of *each bar* is proportional to its corresponding eigenvalue, respectively. A map of PO for the central region is shown. **b** The same orientation map as in **a**. **c** A map of selectivity for the orientation map in **b**, which shows the degree of elongation of receptive fields at each position. Brighter regions represent higher elongation ratios (ER, see Sect. 4). **d**–**f** The same as **a**–**c**, when the columnar grid is randomly displaced with respect to the retinal grid. The displacement is a fixed value of $\delta = 0.75 D$ at random angles

The new model is constructed as follows: Each neuron has a set of random inputs, the distribution of which determines the orientation preference of the target neuron. The retinal position of inputs depends on the position of the neuron on the cortical surface relative to the position of thalamocortical input columns. The columnar structure implies that the density of afferents is higher at the center of columns than between columns. The position of the neuron also determines the distribution of its dendritic arborization and hence the probability of receiving a connection. We assume both distributions to be Gaussian and combine them in a Gaussian probability distribution, localized in the middle between the neuron and the center of the column.^4^ This is an approximation to the overlap of distributions. In terms of receptive fields, this probability distribution describes the sampling from the retinal grid. The density of sampling depends on the distance of the neuron to the column. We fix the total number of samples, and for each column draw a fraction of that number according to the distance (see Sect. 4, for details). Note that the distance is now measured on the cortical surface in the cortical coordinate system.

First, we consider a case where retinal and columnar grids have identical parameters (Fig. 9a). An example for the distribution of connections is shown for a cortical neuron on the top left. The columns that are closest to this neuron are the columns above and below it. As a result, the cloud of samples is elongated in that direction.

To quantify this non-isotropy, we determine the principal axes of the distribution (see Sect. 4). The covariance matrix then indicates a possible elongation of the receptive field, and we take the orientation of the eigenvector corresponding to the larger eigenvalue as the PO. The difference between the larger and the smaller eigenvalue normalized by their sum is used here as a measure of selectivity (see Sect. 4 for details). The orientation map and the map of selectivity obtained in this manner are shown in Fig. 9a–c. This is a map of orientation selectivity as expected from a regular lattice of columns. Note that pinwheels have each orientation represented twice, as expected.

This is not the case, however, if the columnar grid is slightly displaced with respect to the retinal grid (Fig. 9d–f). The rationale for the displacement is again that the competition between ocular dominance columns preserves neighborhoods, represented here by a jitter from the actual position (same as in Fig. 8). The change in the shape of orientation maps, and especially the fact that $360^\circ$ pinwheels now turned into $180^\circ$ pinwheels, can be explained in terms of the change in the position of columns. More specifically, the distance of the nearest columns to each neuron is important in determining its OS, which in turn determines the map. The PO of neurons at each position should therefore be computed from the relative positions of the new columns with the least distances. The resulting map would therefore deviate from the symmetric map of a regular columnar positions. As the symmetry of columns is now broken, $360^\circ$ pinwheels, which need the same distance of all columns in all directions, disappear.

### Monocular versus binocular orientation maps

The maps shown in Fig. 9 are monocular maps. Arranging a different columnar grid for the other eye would result in another orientation map. Therefore, the monocular orientation selectivity at each point on the cortical surface could be different, depending on which eye is actually stimulated.

**Fig. 10:**
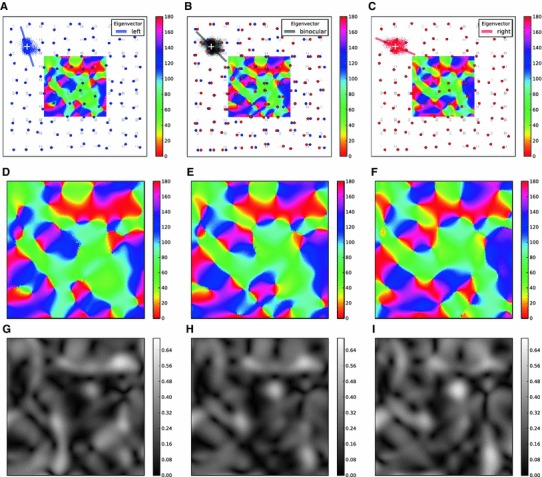
Monocular and binocular orientation maps. **a** Same as Fig. 9d, depicting the grid (*blue circles*), a sample receptive field (*blue cloud*) and orientation map (*center*) for the left eye. Only the orientation of the eigenvector corresponding to the larger eigenvalue is plotted for the sample receptive field (*blue bar*). **b** The binocular orientation selectivity and orientation map are obtained when both grids (for the left and right eye, in *blue* and *red*, respectively) are present. A sample binocular receptive field is shown by the gray cloud. Orientation selectivity is shown by the *gray bar* (eigenvector corresponding to the larger eigenvalue). *c* Same as **a**, for the right eye. The columnar grid (*red circles*) is obtained by random displacement of the retinal grid, independent of the random displacement for the left eye. Only the eigenvector corresponding to the larger eigenvalue is plotted for the sample receptive field (*red bar*). **d**–**f** Map of PO shown for the central region of the grids in **a**–**c**, respectively. **g**–**i** Map of selectivity for the same PO maps in **d**–**f**, respectively. Conventions are the same as in Fig. 9c

Figure 10a, c show the columnar grid and the resultant orientation map for the left and the right eye, respectively. There are overall similarities between the maps of orientation (Fig. 10d, f) and selectivity (Fig. 10g, i), but there are also clear differences. In particular, different maps have different pinwheels. This was also reported in experiments with monocular maps (Hübener et al. 1997).

Under binocular stimulation, inputs from both eyes jointly drive the network. In our model, we assume that each neuron sees the same monocular inputs it received before. The resulting distribution of connections is now a combination of both clouds, shown in Fig. 10b for the sample distribution. The aggregate elongation of receptive fields interpolates between the previous cases, and one obtains a binocular map from these orientations. Again, there are similarities to the respective monocular maps, but they are definitely not identical (Fig. 10e, h).

## Discussion

### Statistics and geometry of orientation selectivity

By combining statistics and geometry of thalamocortical connectivity, we provided a computational model that can simultaneously account for many properties of orientation selectivity and maps thereof observed in the primary visual cortex of species like cats and macaques. We discuss these aspects in the following.

### Size and elongation of receptive fields

Since cortical receptive fields are multi-columnar in our model, they typically span a region larger than a single column on the cortical surface. There is, therefore, no need for a composite receptive field (Braitenberg 1985) to explain why oriented receptive fields are “larger than the mechanism that determines their orientation” (Braitenberg and Schüz 1998). Here, the position of a neuron within the system of columns is important in determining its orientation selectivity, as suggested in Braitenberg (1985), Braitenberg and Schüz (1998). However, unlike in this work, in our model, it is not only the geometric relation of the position with respect to the nearest column that determines the elongation. Rather, the geometric relation to all columns in the neighborhood is important. Specifically, the neuron sees all the columnar receptive fields weighted according to their respective distances.

This feature is a result of the axonal arborization of feedforward projections from thalamus to the cortex that implies a columnar structure. The impact of a column on a neuron’s receptive field is mainly dependent on how close the neuron is to the center of the column. Elongation comes as a result of the non-isotropic localization of the contributing receptive fields, which depends on the exact location relative to the columns.

This has two important consequences. First, it suggests that orientation selectivity is an inter-columnar computation. Inter-columnar computations make particular sense in a sensory modality like vision, where continuous features are being processed. Second, the selectivity of cortical neurons could already be determined by the pattern of feedforward connectivity (Chapman et al. 1991), and hence the neuronal input would be orientation selective even in the absence of cortical activity (Ferster et al. 1996). Note that this would not be the case if orientation was computed in the cortex, for example by the presence of inhibitory neurons in the center of columns. In this case, the feedforward input would be non-oriented and cortical inhibition extracts the preferred orientation out of the non-selective input by cross-orientation suppression.

It should be noted that the elongation of receptive fields in the model is the result of only one type of LGN afferents, as we have not modeled ON and OFF center LGN channels separately. It would be interesting to see how including these two pathways and their interaction change orientation selectivity of neurons and their spatial organization. This is especially important in the context of recent findings by Jin et al. (2011), who demonstrated that the arrangement of ON and OFF subregions provides a more accurate prediction of orientation preference than the overall shape of the receptive field. More specifically, they show that the ON–OFF better predicts the preferred orientation of an orientation column in cats, as compared to ON + OFF. The consequences of incorporating both channels should thus be explored in a more detailed modeling study.

### Relationship between ocular dominance columns and the orientation map

The geometry of cortex induced by the columnar structure of its thalamocortical afferents not only determines the orientation selectivity of a neuron at any given position, but also shapes the selectivity map. Indeed, given the pattern of ocular dominance columns, a unique resultant orientation map follows from our model. This is a strong prediction, as it links the two patterns tightly and inherently together without any further assumptions. Experimental testing of this connection seems also possible.

As the emergence of orientation selectivity and the geometry of its organization rely on the patten of inputs, another consequence of the model is that they could be manifested in any other piece of cortex. No specific structure or geometry is needed at this stage, only the same columnar pattern of thalamocortical afferents must be provided. This might provide an explanation for the result of rewiring studies. They show that redirecting of the visual input to the auditory cortex in ferrets leads to the induction of the same orientation modules as normally in the visual cortex (Sharma et al. 2000). Indeed, rerouted projections from the ipsilateral and contralateral eyes (to the medial geniculate nucleus, MGN) get developmentally segregated in the form of adjacent but non-overlapping eye-specific clusters, similar to the normal retino-LGN projections (Angelucci et al. 1997). Different patterns of input provided to a cortex, therefore, determines its eventual functional specification in our model, rather than some intrinsic difference in the cytoarchitecture or functional properties of the cortex. A similar conclusion has been drawn from the emergence of “barrels” in the transplanted visual (occipital) cortex (Schlaggar and O’Leary 1991).

The link between ocular dominance columns and the orientation map has also the potential to explain other known relationships between the two patterns. First, it has been reported in experiments that the contour lines of the two maps are roughly orthogonal, i.e., iso-orientation lines tend to cross the ocular dominance boundaries at right angles (Hübener et al. 1997; Obermayer and Blasdel 1993; Bartfeld and Grinvald 1992). In our simplified example (Fig. 5), this relation is immediate: The preferred orientation of a neuron is determined by the direction of a line connecting the centers of its nearest columns, which is of course orthogonal to the boundaries of the column. Second, pinwheel centers have been reported to avoid binocular regions (the boundaries between regions of equal ocular dominance), with a tendency to be located at the center of columns (Hübener et al. 1997; Obermayer and Blasdel 1993; Bartfeld and Grinvald 1992). This property is also illustrated in the simplified grid of Fig. 7, where the pinwheels occur at the center of columns.

For more complex patterns of ocular dominance columns (as shown in our Figs. 8, 9, 10), the situation might not be as easy to analyze. It would therefore be interesting to see how the different ocular dominance patterns of different species influence these relations. Of particular interest is the question whether differences in cortical geometry and thalamic projections can explain the characteristic differences between the maps exhibited by different species. It appears that the relationships between the two maps are more precise in monkeys and somewhat more fuzzy in cats. We hypothesize that this might be related to different patterns of ocular dominance in these species, which in turn may reflect different strategies to maintain the retinotopy of binocular inputs.

In our model, the columnar projection of afferents is needed to obtain orientation maps. Therefore, it is also relevant to ask whether orientation maps would be absent in species lacking such columnar pattern. This seems apparent in rodents and lagomorphs, which have neither ODCs nor orientation maps. The presence of ODCs, however, does not seem as clear even in species that do have orientation maps (Horton and Adams 2005). In marmosets, for instance, a primate with orientation maps, a capricious expression of ODC has been reported (similar to squirrel monkeys, see Adams and Horton 2003). While some studies have not found any evidence for ocular dominance (McLoughlin and Schiessl 2006), others suggest a high variability of ODC within the marmoset population, consistent with the studies in other New World monkeys (Roe et al. 2005). In fact, the same controversy existed in squirrel monkeys, “the only primate reported to lack ocular dominance columns” at that time (Horton and Hocking 1996). Later studies in these animals, however, revealed the presence of ODC (Horton and Hocking 1996), although the variability, both within the population and within a single individual, seems to be high (Adams and Horton 2003).

It might be possible, therefore, that in such species (New World monkeys, in particular), as a result of developmental constraints, “structural ODCs” are present, namely the clustered projection of inputs from the eyes to the cortex. The “functional ODCs,” however, need not be expressed, as the segregation of cortical neurons to left-dominated and right-dominated clusters depends on cortical activity. Such a segregation requires that cortical neurons detect the dominance of ocularity in their inputs, when they still overlap during the critical period.^5^ If, for some reason, the initial overlap of innervation from the non-dominant eye is not pruned during this period, and clustered inputs from the two eyes have a persistent overlap, cortical neurons show less segregated responses to monocular stimulations.^6^ In the extreme case of homogeneous overlap of the two clusters, a salt-and-pepper structure of ocular dominance would be expected.

In such a case, we actually expect a smaller displacement in the monocular columnar grids, than what we have assumed here (Figs. 8, 9, 10). The reason is that there would be less competition for keeping a good match with retinotopy, as the afferent projections from the two eyes can now extend their termination to each other’s territory, in an overlapping fashion. The situation would now be more similar to the monocular case described in the discussion of our model (Fig. 7a). In that case, however, we expect observing $360^\circ$-pinwheels in the map. It might be interesting to see whether the presence of such pinwheels in marmosets (McLoughlin and Schiessl 2006) is connected to the variability of ODCs in this species.

It is also tempting to conjecture on the spatial position of CO blobs and its relation to pinwheels. CO staining reveals dense regions (blobs) of higher metabolic activity, which have been reported to correspond to the regions of thalamic terminals and to be centered on ODCs (Horton and Hubel 1981; Livingstone and Hubel 1982). Possibly, they appear at the center of columnar projections of thalamocortical afferents, because the activity is higher in these regions due to stronger synaptic drive. In our model, therefore, CO blobs would coincide with the center of columnar grids. They do not necessarily coincide with pinwheel centers (Bartfeld and Grinvald 1992), though, as these do not necessarily appear at the center of columns (see Figs. 8 or 9 for instance).

### Monocular and binocular maps

Indeed, if the goal is to maintain the ocularity of the afferents, and not to merge them too early into a completely binocular signal, cortex would face a problem: How to optimize the retinotopy of inputs? To achieve this, the number of neighbors for each node needs to be increased. As a consequence, neighborhoods are compromised, in one way or another.

One possibility to attenuate this problem is to employ a probabilistic strategy, where neighborhoods are enhanced in a random fashion. This might exactly be the strategy used in cats, leading to a patchy pattern of ocular dominance columns. An alternative strategy could be a more systematic alignment of columns with the same ocularity, as suggested by the stripy pattern in macaque monkeys. In this work, we have not considered the precise form of this pattern. Rather, we attacked the problem in a generic fashion (a Cartesian grid with some random jitter). To explore the match with the experimental data, more realistic patterns need to be considered.

From our generic model, several conclusions could already be drawn. First, it is possible to obtain orientation maps that are very similar to the ones observed in biology, with the same joint pattern of iso-orientation domains and pinwheel centers. Second, the two monocular maps are different from each other, as their monocular columnar structure is different. Such a difference between monocular maps has also been reported in experiments (Hübener et al. 1997). Moreover, binocular maps are equipped with the same overall structure. There is no need to postulate an extra mechanism to align the monocular inputs of a neuron inducing its binocular selectivity. Although some developmental post-processing might be employed to improve selectivity, the combination of monocular receptive fields is already taken care of while the inputs from different ocular dominance columns are being combined.

The post-processing is, however, even necessary if the monocular selectivities of a binocular neuron are different. In cats and macaques, it has been reported that most of the binocular cortical neurons have the same orientation preference through the two eyes (Hubel and Wiesel 1962; Bridge and Cumming 2001). If the monocular selectivities, carried by the pattern of feedforward thalamocortical afferents to the neuron, were different, a need for a plasticity mechanism would arise, which ameliorates this mismatch during development. Our model thus predicts that binocular neurons have potentially a mismatch in their monocular POs, which decreases during development. Such a developmental process has indeed been reported recently in mouse visual cortex (Wang et al. 2010).

It has been reported in experiments (Gödecke and Bonhoeffer 1996) that the orientation maps established by input from only one eye are very similar. Our model, in contrast, displays different monocular maps. This discrepancy might be due to the fact that the different monocular maps of our model correspond to the mature cortex, where the projections from each eye are already firmly established. During the critical period, however, where connections are generally plastic, it is conceivable that one eye takes over the columns of the other, if the latter has no input (Hubel et al. 1977; Hubel and Wiesel 1977). In this case, a column which was previously dominated by the second eye would now receive predominant input from the first eye. Both eyes have more or less the same receptive fields. To satisfy the latter condition, it is only necessary that retinotopy is preserved for both eyes. This was indeed assumed in our model by resorting to the same retinal grid for different ocular dominance grids (Fig. 10, white squares). If the same retinal input (from the open eye) is now fed to both columns (columns corresponding to the same retinal position), the resulting map would be very similar to the binocular map. The same map would of course also result by changing the roles of the two eyes.

### Retinotopy and orientation maps

It has been reported that retinotopy is not perfect, and visuotopic distortions match the inhomogeneities of the orientation map (Das and Gilbert 1997). The rate of receptive field movement over the cortex is proportional to the corresponding local rate of orientation change on the orientation map. Specifically, the changes in receptive field positions are very abrupt near pinwheel centers, very much related to the abrupt shifts of orientation selectivity near these singularities. We therefore wondered whether our model could provide an explanation for this observation.

As discussed before, the receptive field of a neuron in our model is a multi-columnar feature. Singularities of the orientation map are the points around which these inter-columnar receptive fields change drastically. To illustrate this, consider the simplified hexagonal grid of Fig. 5, which has a singularity in the center (shown in Fig. 7). If one moves away from this center (along the sample receptive field of Fig. 5c for instance), the receptive field changes slowly and smoothly. The reason is that the dominant columns are the same, and moving closer to, or farther away from each column changes the corresponding weights only slightly, and keeps the extent and elongation of the receptive fields more or less the same. This is not the case, however, if one moves over the pinwheel center. Here, although traversing only a small distance, another neighboring column becomes dominant. This leads to a strong change in the receptive field and, as a consequence, to a change in orientation selectivity of the membrane potential. Note that to compute the full spiking response, more details of cortical circuitry and the neuronal model must be included into the model.

The same conclusions would, in fact, be drawn from Fig. 8. Here, moving within an iso-orientation domain changes the receptive field and the orientation selectivity only smoothly: A long distance can be traversed without inducing a significant change in both properties (compare the PO at positions 2 and 3). In contrast, a small displacement around a pinwheel center brings a large change in the extent and selectivity of the receptive field (compare the PO of positions 1 and 2). Again, note that we have not modeled the spiking activity of the neuron within its network; more realistic simulations would yield tuning curves of spiking neurons, which are more selective at the PO and respond with very low rates at the non-preferred orientations. The simple and reduced model considered here, however, demonstrates how the joint inhomogeneity of orientation map and retinotopy could result from the columnar structure.

### Future work

There are several issues that we have not addressed here and that should be accounted for in future work. Devising a more realistic model, in particular, will pave the road to more directly compare our models with biology. It is, therefore, necessary to simulate the model with more biological detail and with realistic parameters of (retinal and cortical) geometry and (thalamocortical) connectivity. It is also necessary to model the spiking responses of neurons within their recurrent network. The latter is indeed the source of the largest portion of inputs a cortical neuron receives (Peters and Payne 1993). This allows one to see the effect of local network operation on the spiking responses, which includes amplification and enhancement of orientation selectivity (for a model of this sort with realistic parameters see McLaughlin et al. 2000).

Orientation selectivity improves during development (Chapman and Stryker 1993; Chapman et al. 1996). We have not considered here any possible developmental mechanisms that are involved in this process. Correlation-based mechanisms like Hebbian synaptic plasticity could be added to the model, in order to study the process of maturation of receptive fields. We have also not explicitly modeled the formation and maturation of ocular dominance columns. A similar mechanism of plastic adaptation could guide this process and govern the competition of columns for cortical territory. The exact pattern of ocular dominance columns was also not considered in our simulations. The exact geometry of this pattern and the consequence of any particular pattern for spatial organization of orientation selectivity is an interesting subject of future research.

Last but not least, we have not considered different types of afferent channels. Receptive field of LGN neurons is either ON or OFF center, i.e., they would respond best to the stimulus if it is a light or dark spot on their centers, respectively. Including both channels into the model would very likely increase cortical selectivity, since they increase the discrimination.

## Methods

*LGN receptive fields.*  In the following, we use $G(r_0,\sigma )$ to refer to a two-dimensional Gaussian centered at $r_0 = (x_0,y_0)$, with isotropic standard deviation $\sigma$

1 $$\begin{aligned} G(r_0,\sigma ) = \frac{1}{2\pi \sigma ^2} \exp {\left( -\frac{(x-x_0)^2 + (y-y_0)^2}{2\sigma ^2}\right) }. \end{aligned}$$

We model the LGN receptive fields, centered at $r^{\mathrm {lgn}}$, as a difference of Gaussians

2 $$\begin{aligned} R_{\mathrm {lgn}}(r^{\mathrm {lgn}}) = G(r^{\mathrm {lgn}}, \sigma _1) - G(r^{\mathrm {lgn}}, \sigma _2). \end{aligned}$$

We normalize the receptive field to a peak value of 1. When an LGN cell is ON center, $\sigma _{\mathrm {on}} = \sigma _1$ and $\sigma _{\mathrm {off}} = \sigma _2$. This is the case in Fig. 2a, and the values are $\sigma _{\mathrm {on}} = 1$ and $\sigma _{\mathrm {off}} = 1.5$. If an LGN cell is OFF center, $\sigma _{\mathrm {on}}$ would be larger than $\sigma _{\mathrm {off}}$, i.e., $\sigma _{\mathrm {off}} = \sigma _1$ and $\sigma _{\mathrm {on}} = \sigma _2$. We have not considered such cells here, though.

*Columnar receptive fields.*  Each column, centered at $r^{\mathrm {col}}_i = (x^{\mathrm {col}}_i, y^{\mathrm {col}}_i)$, is defined by the arborization of $N$ LGN cells. The centers of LGN receptive fields have a Gaussian distribution around the center of a column

3 $$\begin{aligned} r^{\mathrm {lgn}}_i \sim G(r^{\mathrm {col}}_i, \sigma _c), \end{aligned}$$

where $\sigma _c$ determines the dispersion of LGN centers about the center of a column ($\sigma _c = 0.5$ and $N=100$ in Fig. 2b). We draw $N$ LGN centers from this distribution for each column. The $i$-th LGN receptive field is centered at $r^{\mathrm {lgn}}_i$ and is therefore obtained according to Eq. (2) as $R_{\mathrm {lgn}}(r^{\mathrm {lgn}}_i)$.

The receptive field of a cortical neuron in the column, located at $r^{\mathrm {ctx}}_j$, is a combination of these LGN receptive fields. We therefore compute the cortical receptive field as a weighted sum of all LGN receptive fields within a column

4 $$\begin{aligned} R_{\mathrm {ctx}}(r^{\mathrm {ctx}}_j) = \sum _{i=1}^N w_{ij} R_{\mathrm {lgn}}(r^{\mathrm {lgn}}_i), \end{aligned}$$

where $w_{ij} = w(r^{\mathrm {lgn}}_i, r^{\mathrm {ctx}}_j)$ is the weight of $i$-th LGN receptive field in building the receptive field of the $j$-th cortical neuron. This weight summarizes the density of the arborization, which is higher close to the LGN center, and falls off as the distance from the center increases. We approximate the resulting profile again by a Gaussian and model the weight as a Gaussian function of this distance

5 $$\begin{aligned} w(r^{\mathrm {lgn}}_i, r^{\mathrm {ctx}}_j)= G(r^{\mathrm {ctx}}_j - r^{\mathrm {ctx}}_i, \sigma _w). \end{aligned}$$

The resulting receptive field is the aggregate receptive field of the neuron, as a result of integrating all the afferents within the column (samples shown in Fig. 2c, for $\sigma _w = 1$).

Since the shapes of receptive fields are very similar, we idealize them as a “columnar receptive field,” again with a Gaussian profile

6 $$\begin{aligned} R_{\mathrm {col}}(r^{\mathrm {col}}) = G(r^{\mathrm {col}}, \sigma _\mathrm{col}), \end{aligned}$$

where $\sigma _\mathrm{col}$ is an effective standard deviation for the column.^7^ Each neuron on the column would now see the same columnar receptive field, weighted with the distance

7 $$\begin{aligned} R_{\mathrm {col}}(r^{\mathrm {ctx}}_j) = G(r^{\mathrm {ctx}}_j - r^{\mathrm {col}}, \sigma _\mathrm{col}) R_{\mathrm {col}}(r^{\mathrm {col}}). \end{aligned}$$

The Gaussian profile of weights is supposed to reflect the density of arborizations of all LGN afferents, which monotonically falls of with distance to the center. A direct demonstration is given by the simulation in Fig. 2d, where the maximum of the aggregate receptive field (Eq. 4) is plotted for each point on the column, yielding a Gaussian distribution. We take the standard deviation of this Gaussian to be the same as $\sigma _\mathrm{col}$.

For simplicity, we have so far assigned the same position, $r^{\mathrm {lgn}}_i$, to both the center of LGN receptive fields and the center of their axonal arborizations on the cortex. We are effectively assuming that these two coordinate systems are perfectly aligned.

*Hexagonal grid of columns.*  A hexagonal grid of columns is considered in Fig. 5. To obtain the center of columns, we start from a regular grid ($(i,j)$, where $i,j \in \mathbb {Z}$). We then take the coordinates of the hexagonal grid, $(x,y)$, as $x = d \times (i+j)$ and $y = \sqrt{3} d\times (i-j)$. The spacing is determined by $d$, which is $d = 1.5$ in Figs. 5, 6, 7.

As described in the previous section, for each column, a columnar receptive field is assumed. This is a Gaussian localized at the center of the column, with some effective standard deviation ($\sigma _\mathrm{col} = 1.25$ for the example shown in Fig. 5a). The receptive field of a cortical neuron on such a grid is then computed by summing all columnar contributions according to Eq. (7)

8 $$\begin{aligned} R_{\mathrm {ctx}}(r^{\mathrm {ctx}}_j) = \sum _i G(r^{\mathrm {ctx}}_j - r^{\mathrm {col}}_i, \sigma _\mathrm{col}) R_{\mathrm {col}}(r^{\mathrm {col}}_i). \end{aligned}$$

*Orientation selectivity.*  Once we have obtained the cortical receptive fields, we can quantify their orientation selectivity. To this end, we stimulate each neuron with a sinusoidal grating at different orientations

9 $$\begin{aligned} g_{\theta ,\phi }(x,y) = \bar{I} + \varDelta {I} \sin \left( \frac{2\pi }{\lambda } (x \sin \theta + y \cos \theta ) + \phi \right) .\nonumber \\ \end{aligned}$$

Here, $\theta$ is the orientation of the grating, and $\phi = 2\pi f t$ denotes its phase. The spatial frequency is controlled by $\lambda$, and $f$ is the temporal frequency. The strength of the stimulus is given by its luminance, $I$, with $\bar{I}$ and $\varDelta {I}$ being its mean and its modulation, respectively,

10 $$\begin{aligned} \bar{I} = \frac{I_\mathrm{max} + I_\mathrm{min}}{2}, \quad \varDelta {I} = \frac{I_\mathrm{max} - I_\mathrm{min}}{2}. \end{aligned}$$

From this, the Michelson contrast of the grating can be computed as

11 $$\begin{aligned} C = \frac{I_\mathrm{max} - I_\mathrm{min}}{I_\mathrm{max} + I_\mathrm{min}} = \varDelta {I}/\bar{I}. \end{aligned}$$

We choose gratings with maximum contrast, i.e., $C = 100\%$ and $\varDelta {I} = \bar{I}$.

For each orientation, we change the grating phase from 0 to $2\pi$ in steps of $\delta {\phi }$ ($5^\circ$ for Figs. 5, 6 and $10^\circ$ for Figs. 7, 8). The inner product of the grating with the receptive field at each phase determines the input to the neuron

12 $$\begin{aligned} {\mathrm {Input}}(\theta ,\phi ) = \langle R_{\mathrm {ctx}}(r^{ctx}_j) \cdot g_{\theta , \phi } \rangle . \end{aligned}$$

For each orientation, we neglect the mean response and take the modulation (F1 component) of the ${\mathrm {Input}}$ vs. $\phi$ as the Input Modulation. The tuning curve of the neuron, $T(\theta )$, is then obtained by computing the Input Modulation for different orientations of the drifting grating. This we plot in Fig. 5d. Here, and for Fig. 6, the tuning curve is obtained for 18 orientations providing a uniform sampling of the circle, i.e., $\theta = 0, 10, \ldots , 170^\circ$. For Figs. 7, 8, we reduce the number to 8 (steps of $22.5^\circ$), to reduce the simulation time.

From this tuning curve, we obtain the preferred orientation (PO) as the orientation of the maximum response. To quantify orientation selectivity, we compute a global orientation selectivity index (OSI), as $1 - {\mathrm {Circular~Variance}}$ of the tuning curve (Ringach et al. 2002)

13 $$\begin{aligned} {\mathrm {OSI}} = \left| \frac{\sum _\theta T(\theta )\exp (2\pi i\theta /180^\circ )}{\sum _\theta T(\theta )}\right| , \end{aligned}$$

where $\theta$ is given in degrees and $|.|$ denotes the absolute value of the resultant complex number.

The spatial frequency was $\lambda = 0.15$ for all simulations shown here. Tuning this value for different receptive fields, in order to find the best spatial frequency, may increase responses, but does not change the behavior qualitatively (in particular, the PO remains the same, as the model is totally linear).

*Modeling retinal and columnar grids.*  To account for the ocular dominance of columnar grids, we resorted to a simple model: We map the retinal grid (same for both eyes) on the cortex and add a small random displacement to each node to obtain the position of the corresponding ocular dominance column for each eye. This is shown in Fig. 8a. The retinal grid (black circles) is regular with an inter-column distance of 2.5. The columnar grid (crosses) is obtained by adding an independent random jitter to the $x$ and $y$ coordinates of the retinal grid. The jitter in both directions is uniformly and independently sampled from the interval $[-1.5,1.5]$. The extent of the columnar receptive field is the same as before, $\sigma _\mathrm{col} = 1.25$ (shown in Fig. 8a). As before, the same Gaussian describes the weight of receptive fields.

*Extracting RF orientation by PCA.*  The procedure explained so far would be computationally very expensive, if one wanted to simulate a larger grid, at a high resolution. For an orientation map of size $100\times 100$, using for each receptive field, 10 orientations of a grating at 50 phases to map the receptive field, the number of computations would be increased of order $O(10^6)$. The numerical procedure could be many times faster, if a more efficient method to estimate orientation selectivity could be employed. We have therefore used principal component analysis (PCA) in the results shown in Figs. 9 and 10 to expedite the process.

Principal component analysis was used here to extract the principal axis of elongation of a two-dimensional distribution from its covariance matrix. This distribution represents the receptive field of a cortical neuron, which is obtained as follows. For each position $r = (x,y)$, the distance to column centers *on the columnar grid* determines the weight of contribution according to a Gaussian function

14 $$\begin{aligned} w_i = G(r - r^{\mathrm {col}}_i, \sigma _w). \end{aligned}$$

In contrast to previous sections, however, this does not directly weight the columnar receptive field. Instead, it determines the number of samples that are drawn from each column of LGN afferents. This could be considered as “stochastic integration,” which replaces an explicit computation of the covariance matrix from the continuous receptive fields.

The samples are drawn from a joint receptive field, which is a Gaussian centered between the neuron and the column. The rationale is that, if there is a Gaussian distribution describing the density of dendritic arborization of the neuron, and a Gaussian distribution for the density of axonal arborization of afferents within the column, the overlapping distribution could be approximated with another Gaussian, which is centered in between.^8^

The joint distribution of the neuron and the $i$-th column, therefore, can be described as

15 $$\begin{aligned} R = G((r+r^{\mathrm {rtn}}_i)/2, \sigma _r), \end{aligned}$$

where $r^{\mathrm {rtn}}_i$ is the center of the receptive field of the $i$-th column *on the retinal grid*. The extent of the distribution is described by the effective standard deviation, $\sigma _r$.

From this distribution, we sample $n_i$ points ($r^s_k, k=1,\ldots ,n_i$). The number of samples is proportional to the weight ($n_i \propto w_i$): The closer a column is to the neuron on the cortex, the higher the number of samples would be. We normalize the sampling such that a total number of $n$ samples are drawn for each position

16 $$\begin{aligned} n_i \approx \frac{w_i}{\sum _i{w_i}} n. \end{aligned}$$

We have used $n = 10{,}000$ for the results shown here.

Once the samples are obtained, we run a PCA on them to obtain the axis of elongation. We first make a $2\times n$ matrix out of $x$- and $y$-coordinates:

17 $$\begin{aligned} A = \left( \begin{array}{cccc} x^s_1 &{}\quad x^s_2 &{}\quad \ldots &{}\quad x^s_n \\ y^s_1 &{}\quad y^s_2 &{}\quad \ldots &{}\quad y^s_n \end{array} \right) . \end{aligned}$$

We then obtain the covariance matrix:

18 $$\begin{aligned} \hbox {CC} = \left( \begin{array}{cc} c_{xx} &{}\quad c_{xy} \\ c_{yx} &{}\quad c_{yy} \end{array} \right) = \frac{1}{n-1} \bar{A}\bar{A}^T, \end{aligned}$$

where $\bar{A}$ is matrix $A$ after subtracting the mean from each row, and $\bar{A}^T$ is its transpose. CC is now a $2\times 2$ matrix and we compute its two eigenvectors as principal axes of the distribution. The one corresponding to the larger eigenvalue ($v_\mathrm{max}$) is the axis of elongation, which is shown for sample distributions in Fig. 9a, d. We take the orientation of this vector as the PO of the receptive field.

If both eigenvalues are the same, there is no elongation and the distribution is isotropic. The difference of eigenvalues therefore gives a measure of elongation. We take a normalized measure of this difference (normalized by the sum) as an elongation ratio, ER:

19 $$\begin{aligned} {\mathrm {ER}} = \frac{\lambda _\mathrm{max} - \lambda _\mathrm{max}}{\lambda _\mathrm{max} + \lambda _\mathrm{min}}, \end{aligned}$$

which we use here as a measure of orientation selectivity. This returns a value between 0 to 1, corresponding to the minimum and the maximum elongation, respectively.

The retinal grid that is used in Figs. 9 and 10 is a regular grid with spacing $D$. The columnar grid is obtained from this grid by displacing each node. The displacement is fixed to $\delta = 0.75 D$, but its angle, $\psi$, is drawn randomly from a uniform distribution on $[0,2\pi )$, such that

20 $$\begin{aligned} x^{\prime } = x + \delta \cos (\psi ),\quad y^{\prime } = y + \delta \sin (\psi ). \end{aligned}$$

Other parameters are $\sigma _w = \sigma _r = D/2$.

## Footnotes

1 In general, one should be cautious about the co-localization of optical imaging results (pinwheel centers) and anatomy (ODC), as it turns out to be a difficult and not very precise procedure. Localization errors in the range of up to a few hundred micrometers can easily happen (see e.g. Valverde Salzmann et al. 2011). However, a strong correlation between pinwheel centers and ODCs has also been reported when both features were mapped by optical imaging methods (Obermayer and Blasdel 1993).

2 The authors therefore argue for molecular cues, rather than stimulus-driven or spontaneous activity, being responsible for the formation of these columns; a recent study has in fact demonstrated the clustering of heat shock protein 90 alpha in register with ODC of the ipsilateral eye in the developing cat visual cortex (Tomita et al. 2013). Indeed, the total removal of the eyes early in visual development does not impede the segregation of thalamocortical axons into ocular dominance columns, with normal periodicity (Crowley and Katz 1999). However, the result of a more recent study in ferrets (Huberman et al. 2006) shows that preventing stage II retinal waves severely disrupts ODC segregation and patterning, although it does not prevent its formation. The authors therefore suggest that the segregation may already happen when the LGN axons still reside in the subplate (Huberman et al. 2008). Given the existing controversy about the exact mechanism, one may assume that the pattern and segregation of ODC is a combined result of patterned spontaneous retinal activity and axon guidance cues (Calabrese 2009). An argument against a role of spontaneous retinal activity was, however, given by Adams and Horton (2003). Our results in the following do not depend on the exact mechanism responsible for this columnar structure.

3 Note that this is the tuning obtained from the feedforward input alone, before recurrent interactions can modulate it, and before it has been transformed to output activity. The spiking response of neurons in a recurrent network may be different.

4 The result, however, does not change qualitatively, if the probability distribution is centered at the position of neuron (not shown). For further explanation, see Sect. 4.

5 Note that this is now part of ocular dominance “plasticity”, which is governed by neuronal activity. The initial phase of its “establishment” is presumably governed by molecular cues (see Crowley and Katz 2002 for more details on the distinction). Spatz (1989) has also suggested two distinct phases of “formation” and “persistence” for ODCs: “The *formation of ODCs during ontogenesis* in (possibly) all living primates [...] and the *persistence of ODCs throughout life* in a group of monkeys which includes the most advanced species [...] suggest that the formation and the persistence of ODCs are two different developmental steps which probably depend on different mechanisms”.

6 If one eye is deprived, its afferent projections should now overlap less with the clustered input of the other eye, and hence the columnar pattern of the non-deprived eye should be less obscured. As a result, the “hidden” ocular dominance pattern would be more apparent in the deprived animal, as has indeed been reported (DeBruyn and Casagrande 1981; Hess and Edwards 1987).

7 Indeed, since the operation described above “in expectation” amounts to a convolution of two Gaussians, the new distribution is again Gaussian. Its variance is obtained by summing the two partial variances.

8 Note that this was different for the modeling of columnar receptive fields, as the center of a columnar receptive field was assumed to be at the center of the respective column (in Figs. 5, 6, 7, 8). However the results did not change qualitatively, when we repeated those simulations with centers in between the position of a neuron and the center of columns (not shown). Conversely, the results of our simulations here do not change qualitatively, if the probability distribution is centered at the position of neurons (not shown). Orientation maps obtained were exactly the same, and the only difference was at the level of the distribution of random samples. While in the former case (the center of distribution between neuron and column) this distribution was more continuous, in the latter case (the center of distribution at the center of column) the samples drawn were more patchy. This is a natural consequence of the discrete position of columns, which induces a large density of samples around their centers and leaves the regions in between empty. When the position of a neuron is also considered, all these “patches” of samples are shifted toward the neuron’s position, which makes the aggregate distribution of samples more continuous (of course, this also depends on the extent of the receptive fields, and increasing this extent makes the aggregate distributions less discrete). Irrespective of continuity or discreteness, however, the overall orientation selectivity, which is inferred from the relative position of a neuron with respect to its neighboring columns, is the same in both cases.
